# Cytogenetic analysis of the genus *Thoropa* Cope, 1865 (Anura-Cycloramphidae) with evolutionary inferences based on repetitive sequences

**DOI:** 10.1590/1678-4685-GMB-2019-0364

**Published:** 2020-07-06

**Authors:** Luiza Rieder Cholak, Célio F. B. Haddad, Patrícia P. Parise-Maltempi

**Affiliations:** 1 Universidade Estadual Paulista (UNESP), Instituto de Biociências, Programa de Pós-graduação em Biologia Celular e Molecular, Rio Claro, SP, Brazil; 2 Universidade Estadual Paulista (UNESP), Instituto de Biociências, Laboratório de Citogenética Animal, Departamento de Biologia Geral e Aplicada, Rio Claro, SP, Brazil; 3 Universidade Estadual Paulista (UNESP), Instituto de Biociências, Laboratório de Herpetologia, Departmento de Biodiversidade e Centro de Aquacultura (CAUNESP), Rio Claro, SP, Brazil

**Keywords:** FISH, rDNA, snDNA, cytogenetic evolution, chromosomes

## Abstract

Cytogenetics can be a useful tool to assist in taxonomic problems by adding information to the widely used morphological and molecular approaches. These taxonomic problems are especially common in anurans, once they are very diverse, highly polymorphic, and present many cryptic species. The genus *Thoropa* Cope, 1865 is composed of six specialist species that reproduce in rocky outcrops and are distributed throughout the Atlantic Forest and Cerrado ecotones. Phylogenetic studies point to possible cryptic species within the *T. miliaris* group. To assist in the evolutionary and taxonomic understanding of this group, classical cytogenetic techniques were used to find possible molecular markers for the genus through rDNA5S, rDNA18S, and U2snDNA probes and analyze their chromosome distribution in the group of *T. miliaris*. Despite the well conserved karyotype under conventional staining and classical techniques, such as Ag-NOR, our C-banding results showed differences in the centromeric heterochromatin concentration between two populations of *T. miliaris*. Furthermore, some differences among the populations and species were found for rDNA5S and U2snDNA. This study contributes to a better understanding of the evolutionary relationships within the genus; however, the use of different probe sequences, such as satDNA, is essential for a more robust cytogenetic analysis.

## Introduction

The genus *Thoropa* was described by Cope in 1865, and *Thoropa miliaris* was the first species to be named. Since then, five more species have been described and are currently divided into two groups: *Thoropa petropolitana*, with *T. petropolitana* (Wandolleck, 1907) and *T. lutzi* Cochran, 1938 and *Thoropa miliaris*, composed of *T. miliaris* (Spix, 1824), *T. taophora* (Miranda-Ribeiro, 1923), *T. megatympanum* Caramaschi and Sazima, 1984, and *T. saxatilis* Cocroft and Heyer, 1988 (Feio, 2002).

Of the six species that make up the genus, all endemic to Brazil, the two belonging to the *T. petropolitana* group are very rare and have not been seen in the wild for almost five decades (Feio, 2002). Regarding the four *T. miliaris* group species, *T. miliaris* is the most widely distributed, occurring from southern Bahia to the extreme south of São Paulo state, found in humid rocky outcrops at sea level, to altitudes close to 2000 m (Giaretta and Facure, 2004; Feio *et al.*, 2006; Fitzpatrick *et al.*, 2009). *T. megatympanum* is endemic to the Espinhaço mountain range in the Brazilian states of Minas Gerais and Bahia, and inhabits rupestrian fields (Caramaschi and Sazima, 1983; Feio *et al.*, 2002; Eterovick and Sazima, 2004).

The distribution of *T. saxatilis* is restricted to the southern region of Brazil, occurring from Santa Catarina to Rio Grande do Sul states and found on the slopes of Serra Geral mountain range. Finally, *T. taophora*, popularly known as stone frog, sheep frog, or goat frog (Verdade *et al.*, 2009; Brasileiro *et al.*, 2010), inhabits outcrop rocks from the beaches to within the Atlantic Forest on the coastal regions of Serra do Mar mountain range, São Paulo state, similarly to *T. miliaris* (Feio *et al.*, 2002; Brasileiro *et al.*, 2010). In addition, *T. taophora* shows high tolerance to salinity and is commonly found in tidal regions, a niche rarely occupied by amphibians, due to their permeable and non-keratinized skin, which is sensitive to salty water (Abe and Bicudo, 1991; Fitzpatrick *et al.*, 2009).

From a phylogenetic point of view, some studies suggest the existence of cryptic species in the genus, particularly within *T. miliaris* and *T. taophora* (Fitzpatrick *et al.*, 2009; Sabbag *et al.*, 2018), and *T. miliaris* is actually believed to be a species complex (Feio *et al.*, 2006; Sabbag *et al.*, 2018). Sabbag *et al.* (2018), analyzing nuclear and mitochondrial genes for species of the *Thoropa* *miliaris* group, found five distinct clades for *T. miliaris* that appear to be evolving independently, in addition to a paraphyly of *T. miliaris* related to *T. taophora*. The other species in the group appear to be monophyletic.

Morphological changes often do not follow speciation processes (Bickford *et al.*, 2007). In addition, polymorphisms among populations are expected, which requires the use other tools to differentiate species. The biodiversity of anurans must be investigated to allow the development of management plans to ensure the preservation of species, especially the ones of restricted distribution showing evidence of population decline and / or ecological specializations. In addition, a thorough cytogenetic knowledge on groups will set the ground for a better comprehension of chromosomal evolution processes and gene organization.

## Material and Methods

Samples of the four species belonging to the *T. miliaris* group were analyzed: 12 individuals of *T. miliaris* from Paraty, Rio de Janeiro state (RJ) and seven from Santa Teresa, Espírito Santo state (ES); five *T. taophora* individuals from Ubatuba, São Paulo state (SP) and twelve from São Sebastião, SP; four *T. megatympanum* individuals from Santana do Riacho, Minas Gerais state (MG); and five *T. saxatilis* individuals from Timbé do Sul, Santa Catarina state (SC). All fixed specimens were deposited in the Célio F. B. Haddad Amphibian Collection (CFBH), at the Biodiversity Department, Biosciences Institute, UNESP, Rio Claro, SP, Brazil (Table 1). The collection procedures were approved by the Chico Mendes Institute of Biodiversity Conservation (ICMBio) (authorization numbers 55031-2 and 50280-2).

**Table 1:** Species used in the study, with their respective populations, geographic coordinates of collection places, number of individuals of each gender, registration numbers, and gender (M = male; F = female).

Species	Population	Geographic coordinates	Number of individuals	Registration number	Sex
*T. miliaris*	Paraty/RJ	23°13′12.08″S; 44°43′13.54″W	4	CFBH42175; 42177-78; 42205	F
*T. miliaris*	Paraty/RJ	23°13′12.08″S; 44°43′13.54″W	8	CFBH42174; 42176; 42179-82; 42202-03	M
*T. miliaris*	Santa Teresa/ES	19°56′13.64″S; 40°35′53.06″W	1	CFBH43625	F
*T. miliaris*	Santa Teresa/ES	19°56′13.64″S; 40°35′53.06″W	6	CFBH43600-01; 43610; 43613; 43619; 43621	M
*T. taophora*	Ubatuba/SP	23°13′54.46″S; 44°43′02.04″W	2	CFBH42209-10	F
*T. taophora*	Ubatuba/SP	23°13′54.46″S; 44°43′02.04″W	3	CFBH42207; 42211-12	M
*T. taophora*	São Sebastião/SP	23°46′46.63″S; 45°37′08.96″W	2	CFBH43579; 43582	F
*T. taophora*	São Sebastião/SP	23°46′46.63″S; 45°37′08.96″W	10	CFBH43572-74; 43576-78; 43580-81; 43587-88	M
*T. megatympanum*	Santana do Riacho/MG	19°20′53.4″S; 43°59′83.2″W	4	CFBH43594-97	M
*T. saxatilis*	Timbé do Sul/SC	28°49′45.38″S; 49°54′57.44″O	4	CFBH44454-57	F
*T. saxatilis*	Timbé do Sul/SC	28°49′45.38″S; 49°54′57.44″O	1	CFBH44458	M

### Cytogenetic preparations and banding techniques

Cell suspensions were obtained from the liver, spleen, bone marrow, intestine, and testes following a combination of the procedures described by Schmid (1978) and Baldissera Jr. *et al.* (1993) after treatment with 1% colchicine for 4 hours. Conventional 5% Giemsa staining was used for ploidy determination and chromosome morphological characterization. C-banding techniques were performed according to Sumner (1972), with change in barium exposure time and Ag-NOR according to Howell and Black (1980). Five to twenty metaphases were analyzed per individual. All procedures were approved by the Animal Use Ethics Committee (permission 1554/2016), Biosciences Institute, UNESP, Rio Claro, SP, Brazil.

### Base-specific fluorochrome labeling and in situ hybridization

FISH labeling was performed for rDNA18S, rDNA5S, and U2 snDNA probes, the first two from amphibian and the third one from fish, according to Pinkel *et al.* (1986), with modifications according to Cabral de Mello (2010). Telomeric sequence (TTAGGG) FISH followed the protocol provided in the PNA FISH Telomere / FITC kit (Dako Cytomation, Denmark). Probes were amplified from the extracted DNA of the studied species by polymerase chain reaction (PCR) using the primers shown in Table 2 and labeled by PCR or nick-translation using biotin-14-dATP (Invitrogen) or digoxigenin-11-dUTP (Roche, Mannheim, Germany), according to Pinkel *et al.* (1986). Chromomycin A3 (CMA3) technique was performed according Christian *et al.* (1998).

**Table 2:** Probes and primers sequence used in the present work. rDNA 18S and 5S it were designed in this present paper and U2snDNA is from Bueno *et al.* (2013)

Probe	Forward sequence	Reverse sequence	Annealing °C
rDNA18S	AATTCCAGCTCCAATAGCGT	CCGCGGGCCTGATTTGAA	60 °C
rDNA5S	TACGGCCACACCACCCTGAA	CAGGCGGTCTCCCATCCAGGT	59 °C
U2snDNA	ATCGCTTCTCGGCCTTAT	TCCCGGCGGTACTGCAAT	55 °C

### Karyotypic analysis

Corel PHOTO-PAINT X8 was used for the construction of the karyotypes. Chromosomic morphology was visually determined, and the classification was performed according to Guerra (1986). The labeled chromosomes were analyzed using Olympus BX51 photomicroscope (Tokyo, Japan) at 1600x and 2000X magnifications. The best preparations were photographed using a DP71 camera attached to a microcomputer.

## Results

**Figure 1:**
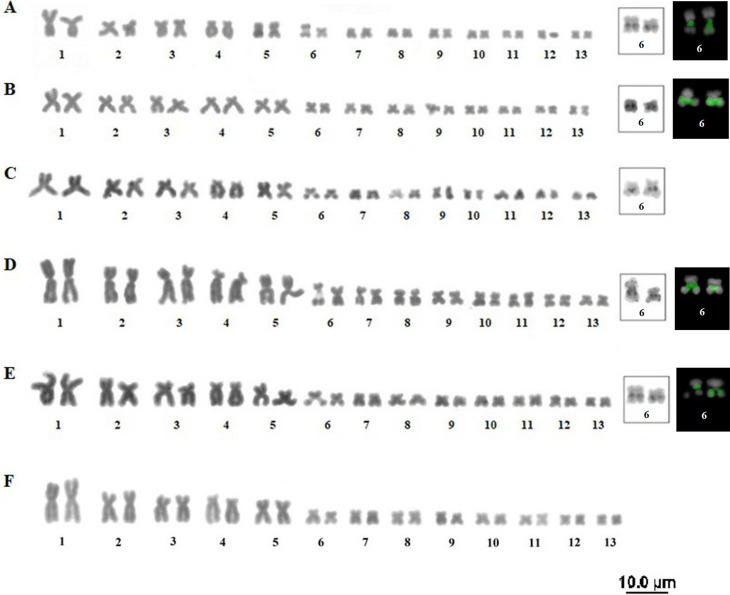
Karyotypes of the four species of the genus *Thoropa*: (A) *T. miliaris*, population of Paraty, RJ; (B) *T. miliaris*, population of Santa Teresa, ES; (C) *T. taophora*, population of Ubatuba, SP; (D) *T. taophora*, population of São Sebastião, SP; (E) *T. megatympanum*, Santana do Riacho, MG; (F) *T. saxatilis*, Timbé do Sul, SC. Highlighted in the white square, the NORs in pair 6, in the black square is the CMA3 marking also in pair 6.

All four species had 2n = 26 and FN = 52 showing a conserved karyotype for the genus, both for ploidy and morphology. All the species / populations showed five pairs of large chromosomes and eight smaller pairs, and all chromosomes were metacentric or submetacentric, with a secondary constriction on the long arm of pair 6 (Figure 1). Regarding the Ag-NOR (Figure 1 – white squares) and CMA3 techniques (Figure 1 – black squares). The results were consistent between the two techniques and all species / populations were marked on the secondary constriction pair.

**Figure 2:**
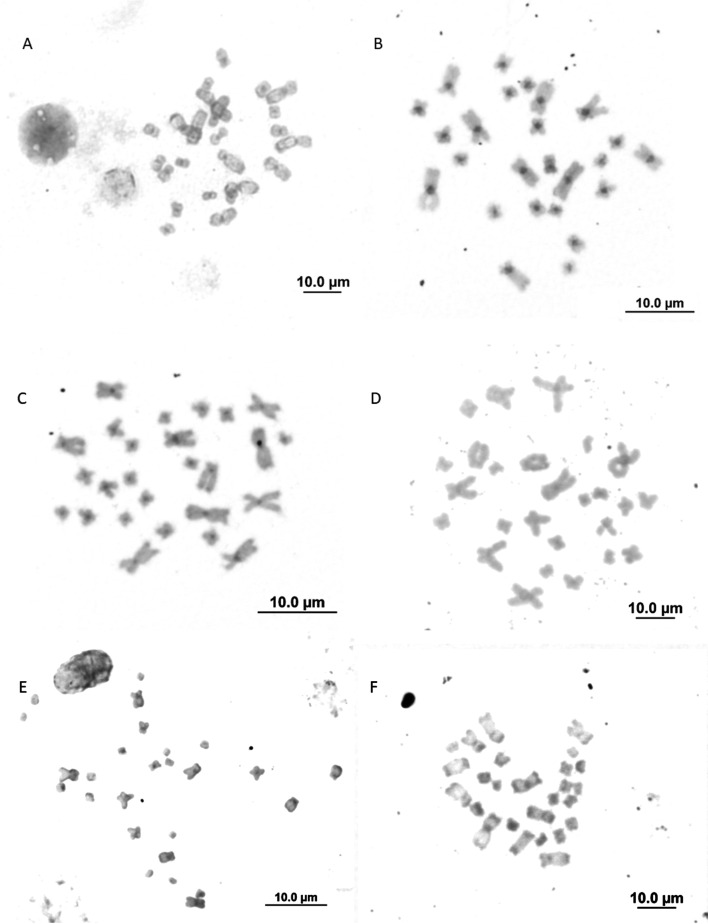
C-banding: *T. miliaris* (A and B) Paraty, RJ and Santa Teresa, ES, respectively; (C and D) *T. taophora* Ubatuba, SP and São Sebastião, SP, respectively; (E) *T. megatympanum*, Santana do Riacho, MG; (F) *T. saxatilis*, Timbé do Sul, SC.

Centromeres and some telomeres markings in chromosomes were observed for all species / populations in the C-banding technique, without interstitial markings (Figure 2). The telomeric probe FISH results for *T. miliaris* from Paraty and Santa Teresa, and for *T. Taophora* from Ubatuba showed no interstitial marking (Figure S1).

**Figure 3:**
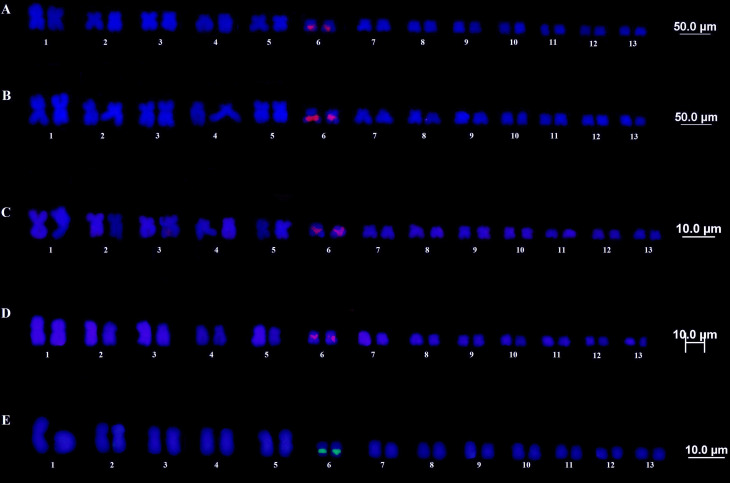
FISH rDNA 18S. (A and B) *T. miliaris*, Paraty, RJ and Santa Teresa, ES, respectively. (C and D) *T. taophora*, Ubatuba, SP and São Sebastião, SP, respectively. (E) *T. saxatilis*, Timbé do Sul, SC.

The FISH technique using a rDNA 18S probe showed marking consistent with those obtained by silver nitrate impregnation, confirming the location of the NOR in the secondary constriction of pair 6 (Figure 3). The same region was also evidenced by the CMA3 technique (Figure 3, black squares).

**Figure 4:**
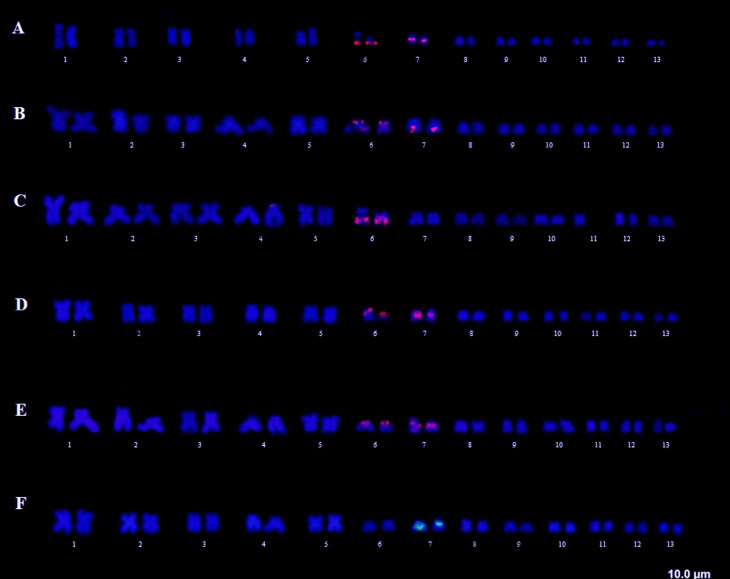
snDNA U2 FISH. (A and B) *T. miliaris*, population of Paraty, RJ and Santa Teresa, ES, respectively; (C) *T. megatympanum*, Santana do Riacho, MG. (D and E) *T. taophora*, population of Ubatuba, SP and São Sebastião, SP, respectively. (F) *T. saxatilis*, Timbé do Sul, SC.

Regarding the U2 snDNA, some signal variations were observed among the analyzed species. In *T. miliaris* from Paraty, signals appeared in the telomeric region of the short arms in pair 6 and in the centromeric region of pair 7. In *T. miliaris* from Santa Teresa, signals were found in these same chromosome pairs and regions; however, the signals of pair 6 appeared in the long arms. For *T. taophora* chromosomes (Ubatuba and São Sebastião, SP), the markings were detected in the telomeric and pericentromeric regions of the pairs 6 and 7, respectively. Similarly, the telomeric region of the long arm of pair 6 in *T. megatympanum* was marked, and, in *T. saxatilis,* only the centromeric region of pair 7 (Figure 4).

**Figure 5:**
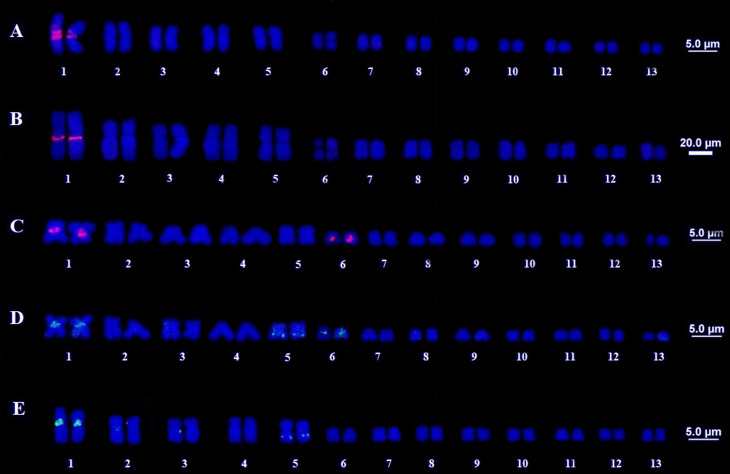
5S rDNA FISH. (A and B) *T. miliaris*, Paraty, RJ and Santa Teresa, ES, respectively. (C and D) *T.* *taophora*, Ubatuba, SP and São Sebastião, SP, respectively. (E) *T. saxatilis*, Timbé do Sul, SC.

*In situ* hybridization using the 5S rDNA as probe showed strong markings in the pericentromeric region of the pair 1 for both *T. miliaris* populations. For *T. taophora*, pericentromeric markings were detected in pairs 1 and 6 of the Ubatuba population, and the same markings in these pairs of the population of São Sebastião, as well as a telomeric signal in pair 5, the latter present only in this population. In *T. saxatilis,* markings were observed in the pericentromeric region of the pair 1 and telomeric region of pair 5 (Figure 5).

## Discussion

Overall, the results point to a conserved karyotype in the group, since the species and different populations presented the same chromosome number (2n = 26), fundamental number (FN = 52) and chromosome morphology, including the presence of a secondary constriction in the long arm of pair 6 (Figure 1). In addition, heterochromatic bands were identified only in telomeres and centromeres (Figure 2), an expected pattern for anurans (Schmid *et al.*, 2014).

Ag-NOR, CMA3 (Figure 1) and rDNA18S (Figure 3) markings confirmed the location of NOR in the secondary constriction of the pair 6 for all the species / populations analyzed. This was also expected, since the presence of only one NOR-containing site is a general characteristic for anurans, considering the ancestral condition of the group (King, 1990). In addition, the heterochromatin concentration differences among *T. miliaris* populations suggest an accumulation of centromeric chromatin in the Santa Teresa, ES population This suggests a possible speciation process (Figure 2B), supported by the recovery of five distinct clades in *T. miliaris*, which may be evolving independently (Sabbag *et al.*, 2018).

The variations observed in U2 snDNA and rDNA5S probes, in turn, allow us to infer some hypotheses for the karyotypic evolution within the group. According to Sabbag *et al.* (2018), *T. saxatilis* would be the oldest species and the first one to diverge, followed by *T. megatympanum* and the *T. miliaris* + *T. taophora* species complex. With specific regard to U2, *T. saxatilis* has a pericentromeric marking in pair 7, similarly to *T. miliaris* and *T. taophora;* however, unlike these, it does not show marking in pair 6 (Figure 4). *Thoropa megatympanum* (the second to differ after *T. saxatilis*) on the other hand, presents only the marking in the telomeric region of the long arms of the pair 6, as well as in *T. miliaris*, population of Paraty. The marking in pair 6 is repeated for all the species and populations, except for *T. saxatilis*; however, in the *T. miliaris* population of Santa Teresa, ES,, and in the two populations of *T. taophora* (Ubatuba and São Sebastião, SP), the marking occurs at the end (telomeric region) of the short arm of pair 6, indicating a possible inversion in the population of Paraty, RJ. Despite having highly conserved sequences in eukaryotes, snDNA U2 vary in number and organization in the genomes of different species (Úbeda-Manzanaro *et al.*, 2010; Garcia-Souto *et al.*, 2015).

Concerning the 5S rDNA, even more variation was observed (Figure 5), since differences were found among species and populations (for *T. taophora*), not only in the position of the marking, but also in the distribution of these sequences among chromosomes. According to Sabbag *et al.* (2018), *T. saxatilis* would have been the first of the group to diverge; however, we cannot consider its karyotypic condition as ancestor. Therefore, it is not possible, with these results, to predict whether the copies of this sequence were acquired in par 6 by *T. taophora* during the differentiation process, or if it was lost in *T. saxatilis*. Both populations of *T. miliaris* maintained markings only in the pair 1, a common feature among species that have 5S rDNA in only one locus (Garcia *et al.*, 2017), having both lost these sequences on chromosomes 5 and 6.

The 5S rDNA consists of approximately 120 bp tandem repeat sequences, highly conserved and flanked by non-transcribed spacer DNA, the latter quite variable in size and sequence identity (Wasko *et al.*, 2001; Eickbush and Eickbush, 2007; Garcia *et al.*, 2017). The 5S rDNA is species-specific and has been used as a good comparative parameter for evolutionary studies at the chromosomal level (Wasko *et al.*, 2001). Polymorphisms involving this sequence are characterized in animal and plant groups (Wasko *et al.*, 2001), including anurans (Vittorazzi *et al.*, 2011), and may be a good marker for distinguishing closely related species, subspecies, and hybrids (Pendas *et al.*, 1995; Rodrigues *et al.*, 2012). Studies with fish (Martins *et al.*, 2002), oysters (Cross *et al.*, 2003), and anurans (Vittorazzi *et al.*, 2011) show that 5S has two smaller units different from each other, which usually appear on other chromosomes than the one where the large unit is located. As sequence homogenization and maintenance is often due to uneven crossing-over or gene conversion (Martins and Galetti, 1999), which occur more frequently in telomeric regions, subcentromeric markings are commonly found in fish (Eirín-López *et al.*, 2012). The number of clusters in which 5S appears organized varies among different groups of plants and animals, and, in fish, 5S is generally clustered into just one chromosomal pair and in this group probably represents the ancestral condition (Martins and Galetti, 1999). Regarding amphibians, urodeles show no intraspecific variation either in number or location of rDNA 5S (Barsacchi-Pilone *et al.*, 1977; Vitelli *et al.*, 1982; De Lucchini *et al.*, 1993), although some studies have reported interspecific variation even among closely related species (De Lucchini *et al.*, 1993). When 5S appears in more than one chromosomal locus, it can represent the different units (larger and smaller) of the gene. In addition, there may be size and / or signal polymorphisms in the clusters between homologs and, possibly, because of the low copy quantity, the signal is weak (Martins and Galetti, 1999). In fact, in fish (Martins and Galetti, 2001) and amphibians (Harper *et al.*, 1983; De Lucchini *et al.*, 1993; Vittorazzi *et al.*, 2011; Rodrigues *et al.*, 2012), unlike the mammalian pattern, 5S may be distributed among several chromosomes. This characteristic may reflect the absence of non-homologous sequence exchange between different 5S-bearing chromosomes, and suggest that these sequences evolve independently (Martins *et al.*, 2002).

Polymorphisms among species / populations were found for snDNA U2 and rDNA 5S sequences. Interestingly, *T. miliaris* populations showed polymorphism with respect to the location of snDNA U2, but not to the location of 5S. According to Sabbag *et al.* (2018), phylogenetic analyses show that *T. miliaris* is probably a species complex, since five clades were recovered for the species, which seems to be evolving independently. Conversely, *T. taophora* populations showed different FISH markings for the rDNA 5S sequence, but had the same markings for snDNA U2; however, it is not possible to make phylogenetic inferences even based on Sabbag *et al.* (2018), as 5S is expected to be polymorphic. These findings provide relevant information to existing molecular and morphological data; however, further cytogenetic approaches, including cytogenomic research, are still needed to better understand these differences and how they may reflect in the group taxonomy.

## Supplementary Material

The following online material is available for this article:

Figure S1 Telomeric probes.
