# Supplementary material for: Cytogenetic analysis of the genus Thoropa Cope, 1865 (Anura-Cycloramphidae) with evolutionary inferences based on repetitive sequences
Source: Genet Mol Biol. 2020 Jul 6;43(3):e20190364. doi: 10.1590/1678-4685-GMB-2019-0364 (PMC7344750; doi:10.1590/1678-4685-GMB-2019-0364)
Supplement: Supplementary file 1 [file 1415-4757-GMB-43-3-e20190364-suppl1.pdf]

Genetics and Molecular Biology 43, 3, e20190364 (2020)

https://doi.org/10.1590/1678-4685-GMB-2019-0364

Supplementary Material to "Cytogenetic analysis of the genus Thoropa Cope, 1865 (Anura-Cycloramphidae), with evolutionary inferences based on repetitive sequences."

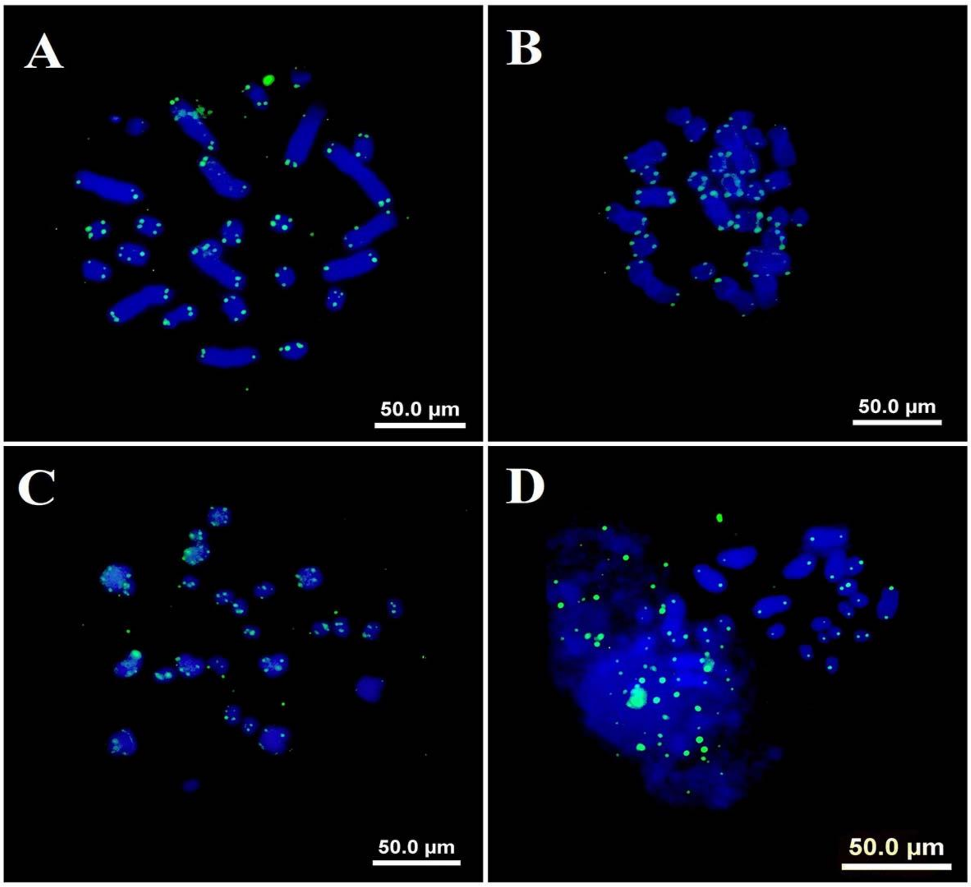

Figure S1 – Telomeric probe. (A) and (B) *T. miliaris*, populations of Paraty, RJ and Santa Teresa, ES, respectively. (C); *T. taophora*, population of Ubatuba, SP; (D) *T. megatympanum*, Santana do Riacho, MG.
